# Amyloid-Like Protein Inclusions in Tobacco Transgenic Plants

**DOI:** 10.1371/journal.pone.0013625

**Published:** 2010-10-26

**Authors:** Anna Villar-Piqué, Raimon Sabaté, Oriol Lopera, Jordi Gibert, Josep Maria Torne, Mireya Santos, Salvador Ventura

**Affiliations:** 1 Institut de Biotecnologia i de Biomedicina and Departament de Bioquímica i Biologia Molecular, Universitat Autònoma de Barcelona, Bellaterra, Spain; 2 Centre for Research in Agricultural Genomics (CRAG) CSIC-IRTA-UAB, Molecular Genetics Laboratory, Barcelona, Spain; University of Crete, Greece

## Abstract

The formation of insoluble protein deposits in human tissues is linked to the onset of more than 40 different disorders, ranging from dementia to diabetes. In these diseases, the proteins usually self-assemble into ordered β-sheet enriched aggregates known as amyloid fibrils. Here we study the structure of the inclusions formed by maize transglutaminase (TGZ) in the chloroplasts of tobacco transplastomic plants and demonstrate that they have an amyloid-like nature. Together with the evidence of amyloid structures in bacteria and fungi our data argue that amyloid formation is likely a ubiquitous process occurring across the different kingdoms of life. The discovery of amyloid conformations inside inclusions of genetically modified plants might have implications regarding their use for human applications.

## Introduction

The intracellular aggregation of polypeptides is a pathogenic feature of cellular degeneration in many human degenerative disorders [1], [2], [3]. Intracellular protein aggregates are formed when misfolded polypeptides accumulate in the cells due to malfunctioning or overloading of the protein quality control machinery or of the components of the degradative pathway [4]. Many disease-associated protein aggregates are composed of filaments known as amyloid fibrils. Amyloid fibrils bind to Thioflavin T (Th-T) and Congo red (CR) due to their repetitive intermolecular β-sheet architecture [5], [6]. It has been shown that the ability to self-assemble into amyloid-like structures is not an unusual feature exhibited by a reduced set of disease-associated molecules with special sequence or structural properties, but rather a property shared by many polypeptides [7]. In addition, the formation of amyloid-like aggregates in living cells is not restricted to animals but has also been observed in fungi [8], [9] and bacteria [10], [11], [12]. Although the formation of amyloids by plant pathogenic bacteria in infected leaves has been recently reported [13], to the best of our knowledge, the formation of amyloid-like deposits in plants by plant-encoded proteins has not been described yet.

The ability to genetically modify plants has allowed the bioproduction of heterologous proteins. In the last decade, plants have become an alternative source for the cost effective production of recombinant polypeptides for therapeutics in animal and human health and diagnostics [14]. The chloroplasts of higher plants are bounded by two envelope membranes that surround an aqueous matrix, the stroma, and the internal photosynthetic membranes, the thylakoids. In chloroplast transformation, and differing from nuclear transformation, the transgene is integrated in the plastid genome via homologous recombination. The flanking sequences of the transformation vector, homologous to the plastid genome, direct the transgene to a specific and unique location without gene silencing, permitting the expression of the desired protein into the chloroplast without needing many generations of gene selection [15]. Transglutaminases (TGases) catalyse post-translational modification of structural proteins by establishing ε-(γ-glutamyl) links and covalent conjugation of polyamines. These proteins are widely distributed in bacteria, animals and plants. Human TGase has been associated to the progression of several neurodegenerative diseases [16]. In plants, this enzyme is poorly characterized and only the maize plastidial TGase gene (*tgz*) has been cloned to date (Patent number WO03102128) [17]. Variants of this TGase have been expressed recombinantly in *Escherichia coli* [18] and *tgz-*transplastomic tobacco plants engineered [19]. Here we use Th-T and CR binding, Fourier Transformed Infrared Spectroscopy (FT-IR) and Transmission Electronic Microscopy (TEM) to study the conformational properties of the protein deposits formed by maize transglutaminase (TGZ) *in vitro* and in the chloroplasts of transplastomic plants, demonstrating that in both cases they exhibit characteristic amyloid features.

## Results

### Maize transglutaminase forms amyloid-like aggregates *in vitro*

We have used two different bioinformatic approaches to detect the presence amyloidogenic regions in TGZ, namely the AGGRESCAN [20] and TANGO [21] algorithms. Both programs coincide to indicate the concentration of aggregation promoting sequences at the C-terminus of the protein. The sequence stretch comprising residues 466–477 is consistently predicted to be a region with high amyloidogenic propensity (Figure S1) suggesting that TGZ might have the capability to aggregate into structures displaying amyloid features.

**Figure 1:**
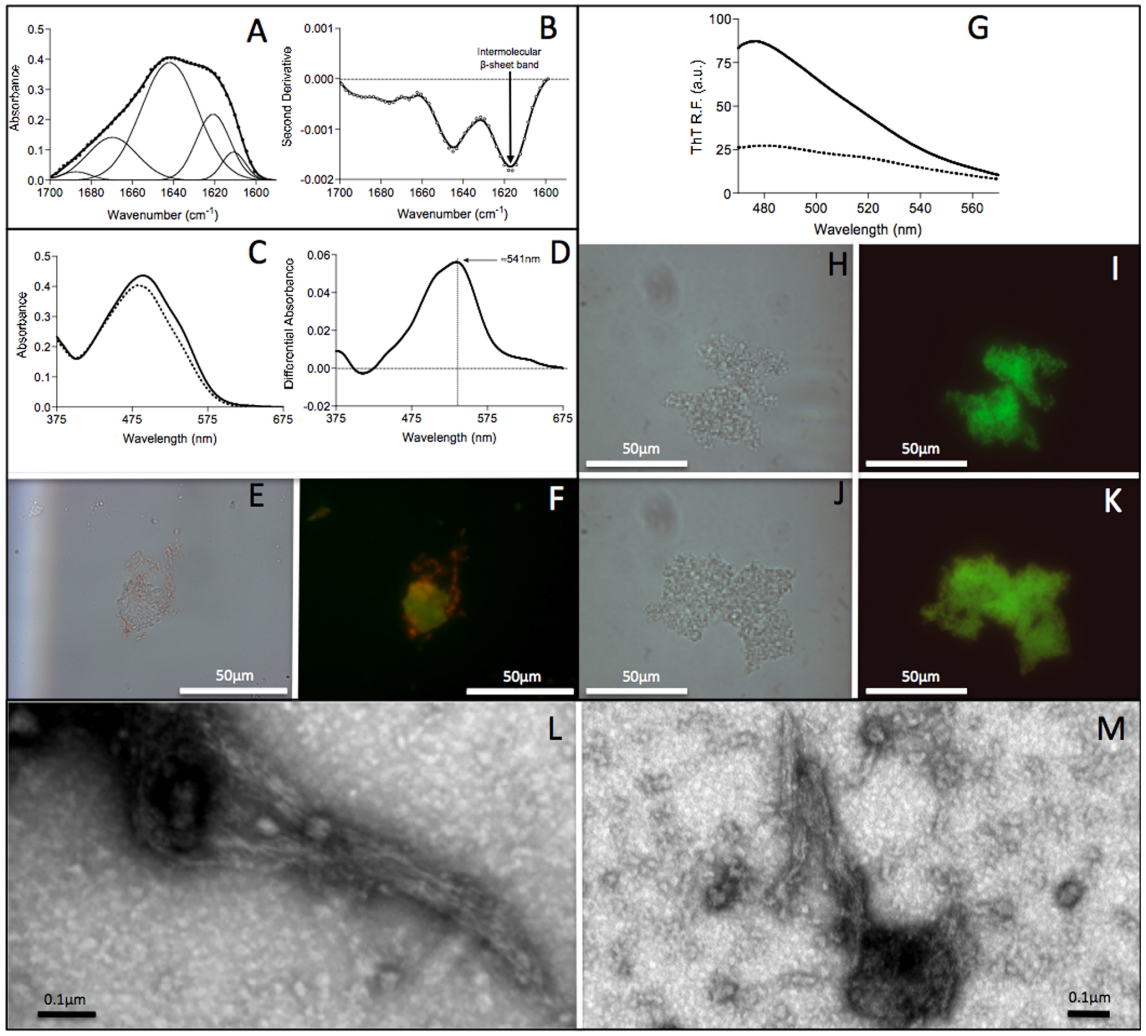
Amyloid-like properties of *in vitro* maize TGZ aggregates. A) Secondary structure of in TGZ aggregates as measured by FTIR absorbance in the amide I region (solid thick lines) showing the spectral component bands (solid thin lines) and the characteristic bands corresponding to intermolecular β-sheet conformations. B) Second derivative of the FTIR absorbance spectra shown in panel A. C) Absorption spectra of Congo red (CR) in the presence (solid line) and absence (dashed line) of TGZ aggregates. Changes in λ_max_ and intensity in CR spectra are observed in the presence of *in vitro* formed aggregates. D) Difference absorbance spectra of Congo red in the presence of TGZ aggregates showing the characteristic amyloid band at ∼541 nm. E) Bright field image of TGZ aggregates stained with Congo red. F) The same image under cross-polarized light showing the characteristic amyloid birefringence. Both images at 40-fold magnification. G) Fluorescence emission spectra of Th-T in the presence (solid line) and absence (dashed line) of TGZ aggregates. The fluorescence intensity increases by three fold in the presence of *in vitro* formed aggregates. H) Bright field image of TGZ aggregates stained with Th-T. I) The same aggregates viewed by fluorescence microscopy under UV light. Both images at 100-fold magnification. J) Bright field image of TGZ aggregates stained with Th-S. K) The same aggregates viewed by fluorescence microscopy under UV light. Both images at 100-fold magnification. L and M) Fibrillar morphology of *in vitro* TGZ aggregates as monitored by transmission electronic microscopy.

TGZ was recombinantly produced in *E. coli*, purified from the insoluble fraction and unfolded in 6 M guanidine hydrochloride. After refolding at 4°C, the protein self-assembles into observable aggregates when incubated at 25°C for one week. The secondary structure content of these aggregates was evaluated by ATR FT-IR in the amide I region of the spectrum (Figure 1A). The second derivative of the absorbance spectrum in this region is dominated by a peak at ∼1618 cm^−1^ (Figure 1B). This signal is typically associated to the presence of intermolecular β-sheet structure. Deconvolution of the absorbance spectrum into its main components (Figure 1A) suggests that this peak arises from the combination of two signals at ∼1610 and ∼1620, both indicative of the existence of short hydrogen bonds between β-strands and compatible with an amyloid like-conformation in the aggregates.

The *in vitro* formed aggregates of TGZ bind to the amyloid diagnostic dye Congo Red (CR) as evidenced by the increase in the absorbance signal and shift of the spectrum towards higher wavelengths (Figure 1C). The different spectrum between the dye in the presence and absence of aggregates allows detecting the characteristic amyloid-like band at ∼541 nm (Figure 1D). In addition, TGZ aggregates incubated with CR display a characteristic amyloid-like green-yellow birefringence when illuminated under cross-polarized light (Figure 1E and 1F).

We further explored the properties of *in vitro* TGZ aggregates by measuring their binding to Th-T. A threefold increase in the maximum emission at 482 was observed (Figure 1G). This change in fluorescence is consistent with TGZ being in an amyloid conformation. The binding of Th-T and the related amyloid dye Thioflavin-S (Th-S) to aggregates was also visualized by using fluorescence microscopy (Figure 1H to 1K). In both cases areas rich in aggregated material were stained with Th-T giving a bright green or green–yellow fluorescence against a dark background.

We monitored the morphology of in vitro formed TGZ aggregates by TEM. The presence of abundant bundles of fibrillar structures with dimensions compatible with an amyloid nature could be observed (Figure 1L and 1M).

### A short C-terminal peptide of maize transglutaminase forms amyloid fibrils

**Figure 2:**
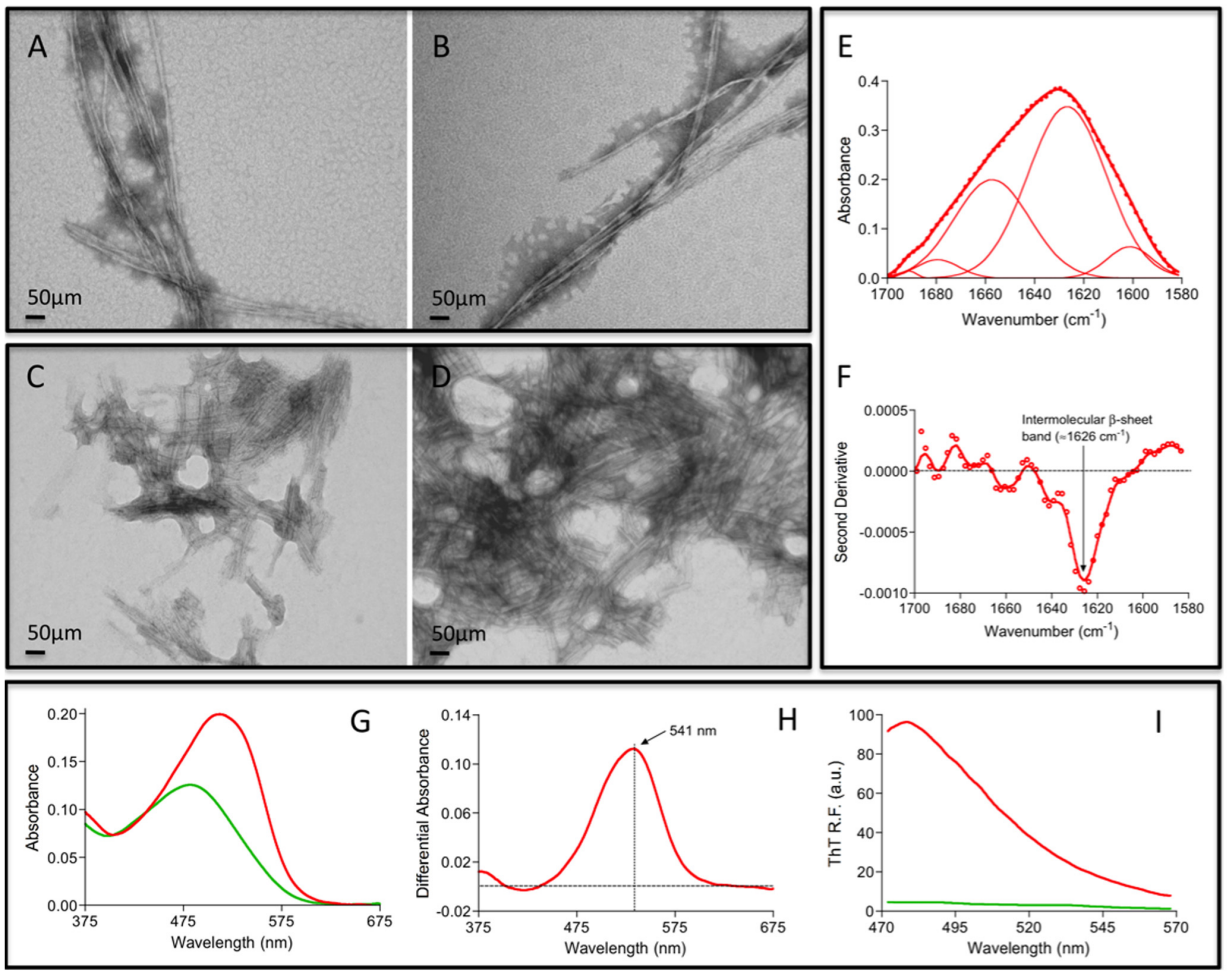
Amyloid-like properties of a C-terminal peptide of TGZ. The peptide QLVVLDILLGKFS corresponds to residues 465–477 of TGZ. A–D) Fibrillar morphology of peptide aggregates formed under quiescence (A and B) and agitation at 500 rpm (C and D) as monitored by transmission electronic microscopy. E–F) Secondary structure of peptide fibrils measured by FTIR absorbance (E) in the amide I region (solid thick lines) showing the spectral component bands (solid thin lines) and second derivative of the FTIR absorbance spectra (F) showing the characteristic bands corresponding to intermolecular β-sheet conformations. G–I) Amyloid specific dyes staining of peptide fibrils. G) Absorption spectra of Congo Red (CR) in presence (in red) and absence (in green) of peptide fibrils. Changes in λ_max_ and intensity in CR spectra are observed in presence of fibrils. H) Difference absorbance spectra of Congo red in the presence of peptide fibrils showing the characteristic amyloid band at ∼541 nm. I) Thioflavin-T (Th-T) fluorescence emission spectra in the presence (in green) and absence (in red) of peptide fibrils. The fluorescence intensity increases by 20-fold in the presence of these aggregates.

Polypeptide sequences might contain local regions with high aggregation propensity that can nucleate the early steps of aggregation [22], [23]. As described above, the region comprising residues 466–477 at the C-terminus has the highest predicted aggregation propensity in the TGZ sequence. To assay if this region has the ability to self-assemble and act as possible nucleation element in the aggregation process of TGZ we synthesized and characterized the amyloidogenic properties of the peptide QLVVLDILLGKFS corresponding to TGZ residues 465–477 (Glu-465 was included to provide solubility to the peptide during its synthesis). The peptide was incubated in 50 mM TRIS at pH 7.5, 150 mM NaCl at 25°C for 48 h at 100 µM in quiescent or agitated conditions. In both cases the formation of fibrillar structures with size and morphology compatible with an amyloid nature could be observed by TEM. Fibrils formed under quiescent conditions were longer (Figure 2A and 2B) than those in agitated samples, which tend to cluster together (Figue 2C and 2D). We analyzed the secondary structure content of quiescent fibrils by ATR FT-IR in the amide I region of the spectrum (Figure 2E and 2F). The second derivative of the absorbance spectrum in this region is dominated by a peak at ∼1626 cm^−1^ (Figure 2F) confirming the presence of intermolecular β-sheet structure in the fibrils. Deconvolution of the absorbance spectrum into its main components (Figure 2E) results in five main signals corresponding to the presence of extended β-sheets (1604, 1628 and 1692 cm^−1^) and turns (1660 and 1680 cm^−1^) compatible with an amyloid conformation of the peptide inside the fibrils. The strong changes promoted by the fibrils in the absorbance spectrum of CR (Figure 2G and 2H) and the fluorescence spectrum of Th-T (Figure 2I) confirm the amyloidogenic properties of the fibrillar structures formed by the most aggregation-prone sequence of TGZ.

### Maize transglutaminase forms amyloid-like aggregates in the leaves of transplastomic tobacco plants

**Figure 3:**
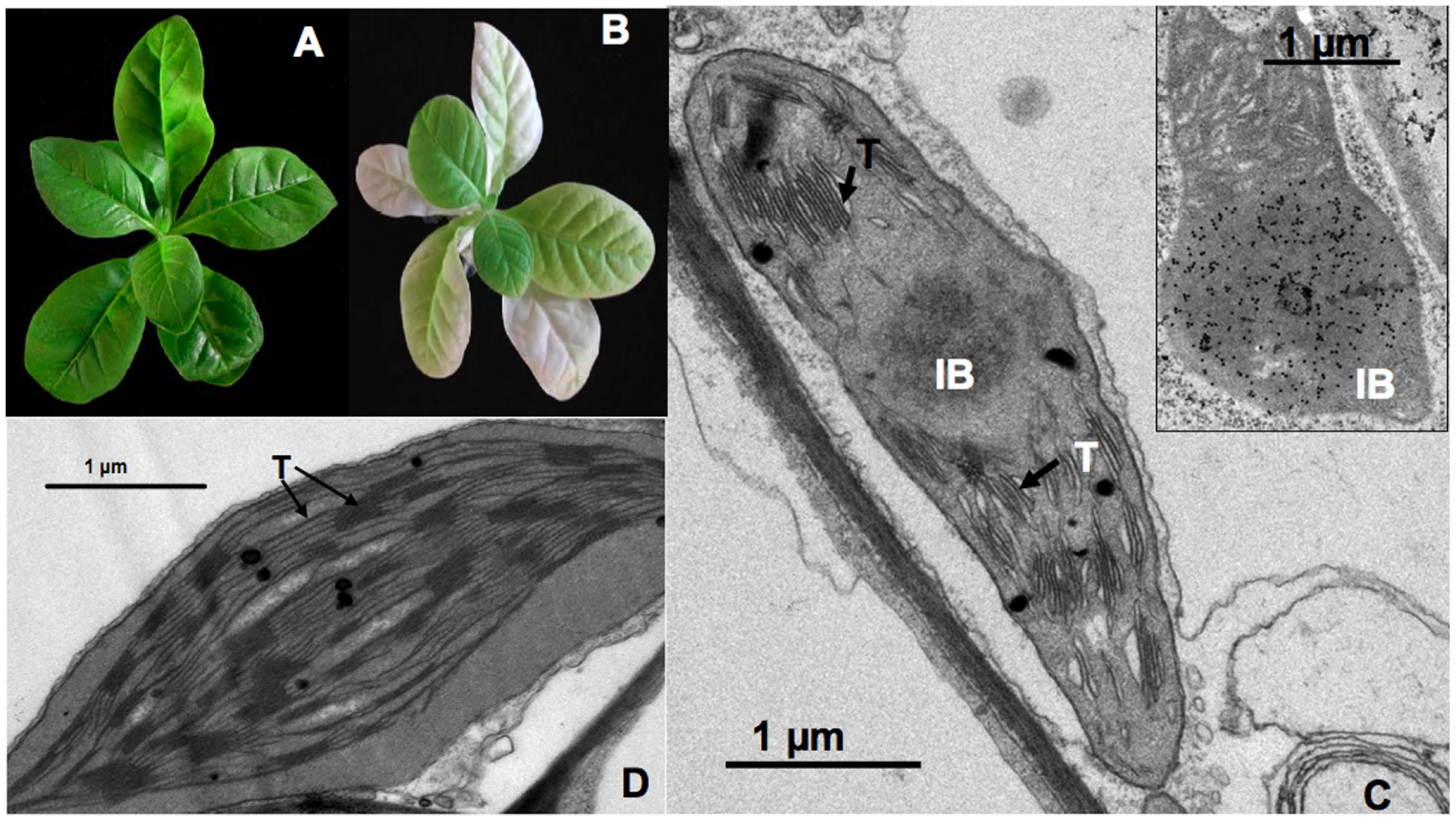
Maize TGZ forms protein inclusions in transplastomic tobacco chloroplasts. A) Aspect of wild type tobacco plant. B) Aspect of *tgz*-transplastomic tobacco plant. C) TEM image of a *tgz*-transplastomic tobacco chloroplast. A high number of appressed thylakoid membranes (arrows), membrane interruptions and IB presence are shown. Inside: subcellular immunolocalization of TGZ protein in the IB of a tobacco *tgz-*transformed chloroplast using an anti-TGZ antibody (1:3000) (see M & M). D) TEM image of a WT tobacco chloroplast showing normal thylakoid membranes and interconnexions. IB, inclusion body; T, thylacoids.

Homoplasmic tobacco *tgz-*transgenic plants presented abnormal phenotype with respect to the leaf colour, having pigment deficiencies (Fig. 3A) and thylakoid appression abnormalities (Figure 3B). The TGZ protein was immunolocalized into chloroplast inclusion bodies (Figure 3C insert), suggesting that in the plant the protein is present in an at least partially aggregated state, which might coexist with functional conformations as shown for bacterial inclusion bodies [24]. The thylakoids in the chloroplasts of non-transgenic plants displayed a normal arrangement with grana stacks consisting of 15–20 tightly appressed thylakoid membranes interconnected by stroma thylakoids (Figure 3D).

**Figure 4:**
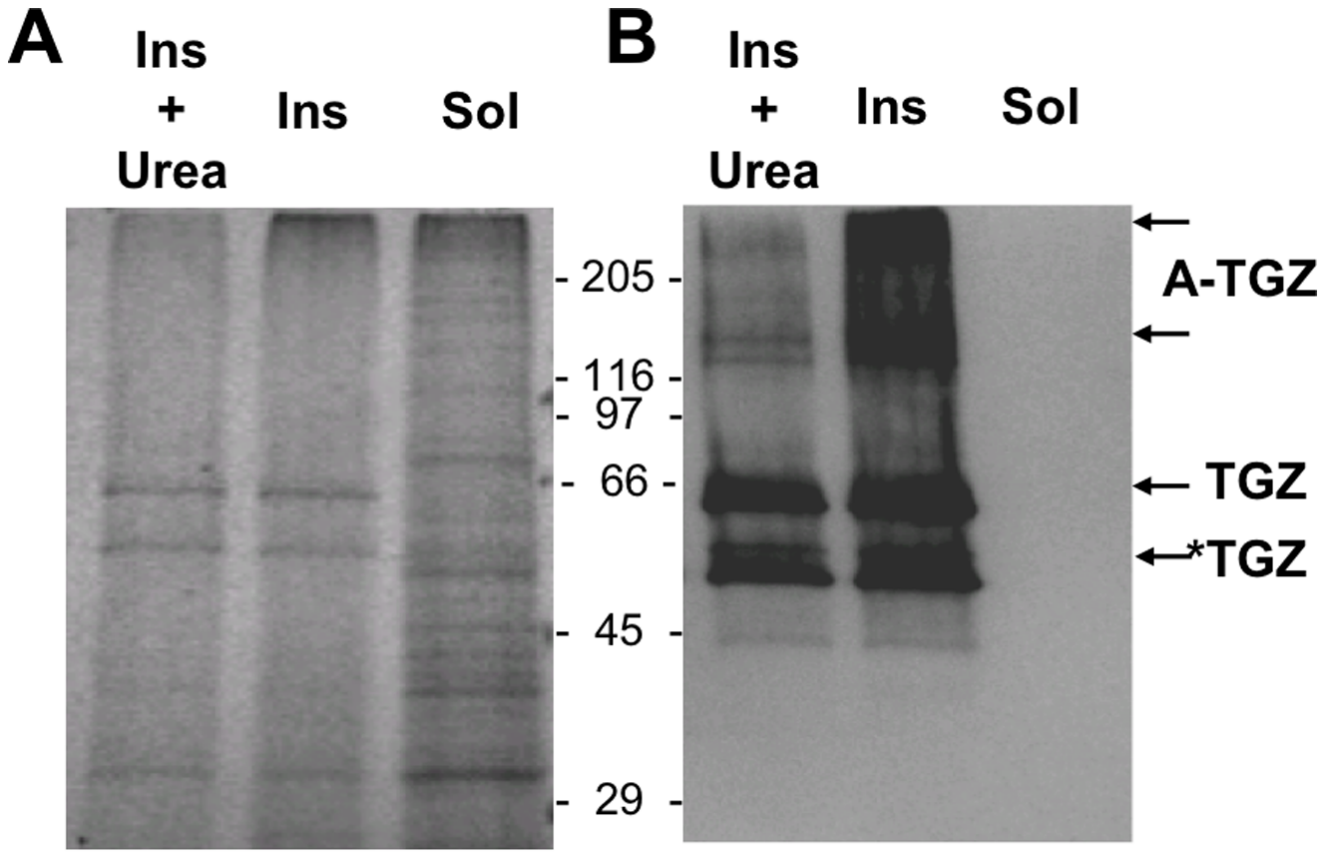
Localization of maize TGZ in the insoluble fraction of leaves in transplastomic tobacco plants. A) SDS-PAGE analysis of the soluble (Sol) and insoluble fraction of transplastomic leaves before (Ins) and after 8 M Urea incubation (Ins + Urea). B) Western Blot of the soluble (Sol) and insoluble fraction of transplastomic leaves before (Ins) and after 8 M Urea incubation (Ins + Urea) using a polyclonal antibody raised against maize TGZ protein (see M & M). Three main types of TGZ species are detected: *TGZ corresponds to a truncated species, TGZ to the full-length protein and A-TGZ to SDS-resistant aggregated forms. The amount of A-TGZ decreases significantly in the presence of 8 M Urea. The positions of molecular weight markers are indicated (in kDa).

We analyzed the protein content of the soluble and insoluble fractions of transgenic plants by SDS-PAGE and Western Blot using an anti-TGZ antibody to determine if TGZ is effectively found in an aggregated state *in vivo* (Figure 4). In spite of the much higher protein content of the soluble fraction, TGZ is absolutely absent in this fraction and localizes exclusively into the insoluble fraction, in which constitutes a major protein component. Three different types of TGZ bands are detected by Western Blot in the insoluble fraction upon SDS-denaturation: a first band corresponding to a truncated species, according to its smaller size when compared with purified TGZ, a second band corresponding to the full length monomeric protein and several intense high molecular bands corresponding to SDS-resistant aggregated species. This SDS-resistant species resemble the oligomeric species found in aggregated solutions of amyloid proteins like Aβ-peptide [25]. Like in the case of amyloid assemblies, in addition to SDS, high chaotropic reagent concentrations are required to disrupt these aggregated species, indicating that they are stabilized by strong intermolecular interactions.

**Figure 5:**
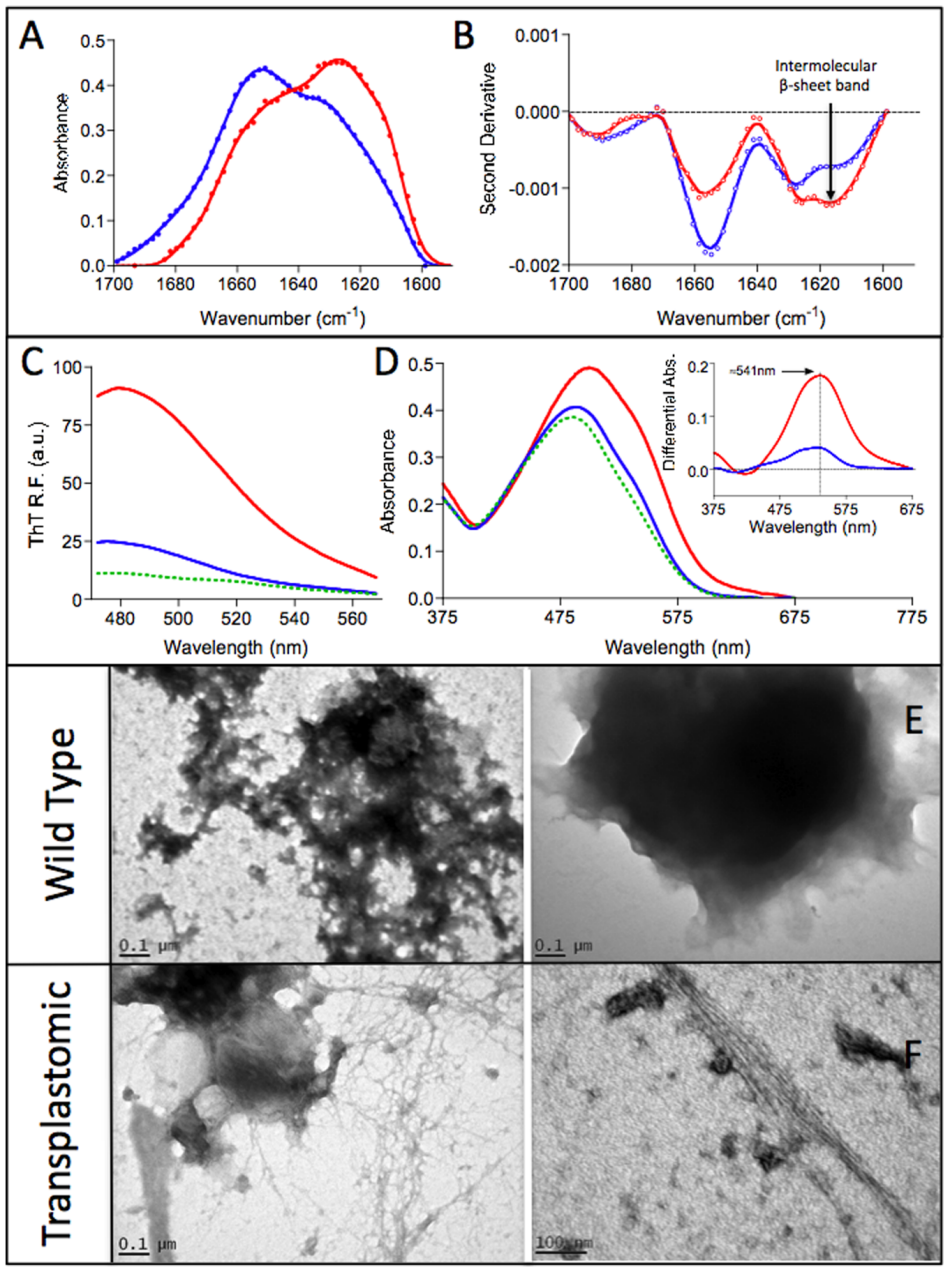
Amyloid-like properties of transplastomic tobacco protein aggregates. A) Secondary structure of transplastomic (red) and WT (blue) plants protein aggregates as measured by FTIR absorbance in the amide I region. B) Second derivative of the FTIR absorbance spectra shown in panel A. C) Fluorescence emission spectra of Th-T in the absence (green) and presence of transplastomic (red) and WT (blue) plants protein aggregates. The fluorescence intensity increases in the presence of protein aggregates. D) Absorption spectra of Congo red (CR) in the absence (green) and presence of transplastomic (red) and WT (blue) plants protein aggregates. Changes in λmax and intensity in CR spectra are observed in the presence of protein aggregates, in the inset it is shown the difference absorbance spectra of Congo red in the presence of transplastomic (red) and WT (blue) plants protein aggregates, showing the characteristic amyloid band at ∼541 nm. E and F) Morphology of transplastomic and WT plants protein aggregates as monitored by transmission electronic microscopy.

To analyze if the aggregates formed by TGZ in transplastomic tobacco plants display amyloid features similar to those observed *in vitro* we isolated the protein insoluble fraction. The same amount of WT tobacco plant leaves were fractionated and analyzed simultaneously as a negative control. The ATR FT-IR spectrum in the amide I region of transplastomic aggregates is significantly different from that of WT aggregates (Figure 5A). The spectrum of transplastomic aggregates is dominated by an intermolecular β-sheet band at ∼1620 cm^−1^ whereas that of WT plants presents a major band at ∼1656 cm^−1^ associated to unstructured and/or α-helical conformation. The second derivative of the spectrum confirms that the intermolecular β-sheet is the main secondary component of transplastomic protein aggregates (Figure 5B).

Consistently with a β-sheet enriched architecture, transplastomic protein aggregates bind to Th-T (Figure 5C) and CR (Figure 5D) promoting the expected spectral changes for amyloid-like structures. Although WT aggregates also exhibit some binding to these dyes the spectral changes they promote are much weaker that in the case of transgenic plants (Figure 5C and 5D).

We further analyzed the morphology of both types of protein deposits by TEM. In both cases we could detect the presence of aggregates. However, they display different structural properties. Whereas, in most of the transplastomic samples the presence of abundant fibrillar material could be observed (Figure 5F), these types of structures are absent in WT samples and their aggregates appear as amorphous material (Figure 5E). In addition, the presence of isolated bundles of fibrillar structures with dimensions and morphology compatible with amyloids could be observed in transplastomic aggregates (Figure 5F) while despite the analysis of large number of fields we could not observe such fibrils in WT samples and its aggregates appear to lack any regular structure (Figure 5E).

## Discussion

In the present work we show that maize TGase (TGZ) displays an intrinsic propensity to form aggregates displaying amyloid-like features when it is refolded *in vitro* in the absence of the components of the protein quality control machinery. The C-terminal TGZ sequence stretch comprising residues 465–477 is able to form highly ordered amyloid fibrils and may act as trigger of the aggregation process. *In vivo*, at any given time, the concentration of a protein in its native state results from a balance between the rate of protein synthesis, the rate of de novo folding, the stability of the protein conformation and the degradation rate [26]. The continuous and high translation rates occurring during the expression of proteins in transplastomic plants tends to unbalance this equilibrium, saturating and/or de-coordinating the mechanisms to assist the process of protein folding or the degradation of misfolded species, which ultimately would result in the accumulation of a significant population of non-native conformers, that in the case of TGZ, due to its intrinsic tendency to aggregate, accumulate as insoluble inclusion bodies into the chloroplast. These aggregates consist, at least in part, of fibrillar material displaying amyloid-like features. Although TGZ species appears as major component of the insoluble fraction in the leaves of transplastomic plants, the possibility that the observed amyloid structures would contain other endogenous plant proteins that co-aggregate with TGZ as observed for protein aggregates formed in bacteria [27] or in mammalian cells [28] cannot be discarded. The saturation of the folding machinery and degradative pathways by misfolded TGZ species as well as the refractivity of TGZ aggregates to proteolysis might be all factors contributing to the accumulation of the observed insoluble material. The presence of truncated forms of TGZ in insoluble fraction of transgenic plant leaves suggests that the proteolytic cellular response targets misfolded TGZ species. However, the role of the different components of the protein quality control machinery in the formation of amyloid-like conformations should be addressed in depth before we can assemble a more precise picture of the process of protein aggregation in plant cells.

To the best of our knowledge, the present report describes for the first time the formation in plants of protein aggregates sharing structural features with the protein deposits associated to human disorders like Alzheimer's or Parkinson's diseases. The formation of amyloid-like aggregates has been recurrently observed in animal cells [1], [29], described in fungi [9] and more recently in bacteria [24], [30]. Our data indicate that also in plants the accumulation of misfolded proteins after their synthesis at the ribosome might trigger their self-assembly into ordered β-sheet enriched macromolecular structures and therefore suggests that amyloid formation is an omnipresent process occurring across all the kingdoms of life.

The accumulation of TGZ in the chloroplasts has dramatic effects for the plant phenotype. In a first developmental phase, TGZ over-expression promotes an increase in the granum size (i.e. increase of the number of stacked thylakoids) with a concomitant decrease of stroma thylakoids and some impairment in the photochemistry of photosynthesis [19], finally the extended effect of this over-expression result in increased oxidative stress symptoms and progressive cell degeneration [31]. Although initially this phenotype was attributed to an increase in the TGZ activity, a more generic toxic effect of the amyloid containing aggregates formed by TGZ on chloroplast membranes cannot be discarded. Accordingly, Aβ-peptide, α-synuclein, and prion proteins are known to promote the formation of distinct amyloid structures that destabilize and disrupt membrane structures [32] and cellular oxidative stress constitutes a common characteristic of amyloidogenic disorders including Alzheimer's disease [33]. Interestingly enough, the expression of Aβ-peptide in transgenic rice plants has been shown to promote significant endoplasmic reticulum (ER) stress suppressing the synthesis and targeting of secretory proteins including storage proteins during seed development, resulting in alternation of grain phenotype and changing the expression of genes and proteins [34]. Although the aggregated state of Aβ-peptide was not evaluated, the data in this study together with these in the present work suggest that the expression of aggregation-prone proteins in transgenic plants may have a generic detrimental effect in cell homeostasis. Despite no reports exists to date, in light of these data the existence of plant disorders associated to the aggregation of natural endogenous proteins cannot be completely discarded.

As in the case of bacteria, the formation of heterologous protein aggregates is a frequent observation during bioproduction in transgenic plants [35], [36], [37]. Importantly, the inclusion bodies formed in bacteria by proteins unrelated to any human disease have been shown to be toxic for mammalian cells [38], supporting the view that general mechanisms appear to underlie the cytotoxicity of many amyloid-like protein aggregates for mammalian cells independently of their source or sequence [39]. In addition, the uptake of heterologous amyloid-like material has been recently suggested to be a factor of risk for the initiation of human amyloid diseases [40]. The extent to which the presence of amyloid-like conformations is a common or anecdotic characteristic of protein deposits in genetically modified plants should be further studied for other polypeptides, tissues and species. In any case, the present data argue that the conformational properties of individual recombinant proteins produced in transgenic plant systems should be investigated in depth before its use for animal or human applications.

## Materials and Methods

### Protein expression in *Escherichia coli* and purification

*E. coli* strains DH5α and BL21(DE3) were used as cloning hosts for construction of expression plasmids and for protein expression, respectively. For protein expression, transformed (pET28-TGZ) *E. coli* BL21colonies were grown in LB medium containing 30 µg kanamycin/ml to an OD_600_ of 0.4 induced for 3 h with 0.4 mM IPTG, and finally harvested by centrifugation [18]. Intracellular recombinant proteins were released with CelLytic BII reagent (Sigma). TGZ was expressed as a histidine-tagged fusion and purified from the insoluble fraction under denaturing conditions (20 mM TRIS at pH 8, 0.5 M NaCl, 20 mM imidazole and 6 M GndHCl) by affinity chromatography on FF-Histrap histidine-tag resin (Amersham). Buffer was exchanged by gel filtration on Sephadex G-25 column (Amersham) with 50 mM TRIS at pH 7.5, 150 mM NaCl at 4°C.

### *In vitro* protein aggregation

TGZ fibrils were assembled at 25°C at a final protein concentration of 20 µM. TGZ aggregation was followed by measuring the transition from the soluble to the aggregated state by UV/Vis spectrophotometry at 350 nm. After a week, the fibrillation reaction was considered to be finished since no further increase in the scattering signal was observed.

### Plant transformation

To transfer the maize (*Zea mays*) *tgz gene* [17] to tobacco chloroplasts, the gene was PCR amplified, fused to the promoter and 5′untranslated region of the *psbA* gene and then introduced into the multiple cloning site of pAF [15], to give the final vector, pAF*- tgz13* [19]. Plant regenerants analysis was performed as previously described [19]. After transplanting homoplasmic *tgz-*transgenic plants were not able to set seed, and died, so were maintained *in vitro* [19].

### Extraction of insoluble protein fraction from tobacco leaves

To extract the insoluble protein fraction from tobacco leaves, transformed and untransformed leaves from *in vitro*-grown plants were ground in liquid nitrogen and 100 mg resuspended in 7 vols. of protein extraction buffer consisting on 20 mM Tris-HCl, 150 mM NaCl, pH 7.5, plus protease inhibitors. The homogenate was filtered through two layers of cheesecloth and 3 µL of DNase I and RNase from 1 mg/mL stock (25 µg/mL final concentration) and 3 µL of 1 M MgSO4 were added and the resulting mixture was further incubated at 37°C for 30 min. Protein aggregates were separated by centrifugation at 10 000 × g for 15 min at 4°C. Finally, the aggregates were washed with the same buffer containing 0.5% Triton X-100 and twice with sterile PBS. After a final centrifugation at 10 000 × g for 15 min, pellets were stored at −20°C until analysis. The frozen pellets were reconstituted in 50 mM TRIS at pH 7.5, 150 mM NaCl.

### TEM observations

Tobacco leaf thin sections (less than 0.5 mm) were fixed by vacuum infiltration with 2% paraformaldehyde and 2.5% glutaraldehyde in 0.1 M phosphate buffer pH 7.4. After washing, they were fixed in osmium tetroxide for 2 h in the same buffer, dehydrated through an acetone series and embedded in Spurr resin by infiltration. The blocks were polymerized for 48 h at 60°C. Ultrathin sections were obtained with an Ultracut UCT ultramicrotome (Leica) using a diamond knife, and mounted on gold grids (200 mesh). To immunolocalize subcellularly TGZ into tobacco chloropolasts, leaf slices were fixed with 4% paraformaldehyde and 0.5% glutaraldehyde in 0.1 M phosphate buffer (pH 7.4) for 2 h at 4°C. After washing, samples were dehydrated through an ethanol series and embedded in Lowicryl K4M resin (Pelco International, Redding, Calif., U.S.A.) at -35°C. Blocks were polymerized under a UV lamp at −20°C for 24 h and ultrathin sections were mounted on gold grids. The primary antibody AbTGZ4 [18] was used at 1:5.000 dilution and a solution of 12 nm diameter colloidal gold-affinipure anti-mouse IgG (Jackson Immunoreserach) diluted at 1:30 in the blocking solution was used as the secondary antibody. Control samples were treated only with blocking solution or pre-immune serum following the same protocol. The sections were examined under a Jeol-JEM-1010 transmission electron microscope at 80 kV.

### FT-IR spectroscopy

Attenuated total reflectance (ATR) FT-IR spectroscopy analyses of purified insoluble protein fractions of bacteria and plant leaves were performed using a Bruker Tensor 27 FT-IR Spectrometer (Bruker Optics Inc) with a Golden Gate MKII ATR accessory. Each spectrum consists of 20 independent scans, measured at a spectral resolution of 1 cm-1 within the 1800–1500 cm-1 range. All spectral data were acquired and normalized using the OPUS MIR Tensor 27 software. Second derivatives of the spectra were used to determine the frequencies at which the different spectral components were located. Infrared spectra were fitted overlapping Gaussian curves and the amplitude, centre and bandwidth at half of the maximum amplitude and area of each Gaussian function were calculated using a non-linear peak fitting program (PeakFit package, Systat Software, San Jose, CA, USA).

### Congo Red Assay

CR interaction with the purified protein fractions of bacteria and plant leaves was tested using a Cary100 (Varian Inc., Palo Alto, CA, USA) UV/Vis spectrophotometer by recording the absorbance spectra from 375 nm to 675 nm using a matched pair of quartz cuvettes of 1 cm optical length placed in a thermostated cell holder at 25°C. Final CR and protein concentrations were 10 µM and 0,04 mg/ml in BUFFER. Spectra were recorded after 2 min equilibration and solutions without protein and solutions without Congo red were used as negative controls. Binding of CR to a 10 µM amylin amyloid fibril solution was used as positive control. For optical microscopy analysis, proteins were incubated for 1 h in the presence of 50 µM CR. After centrifugation (14 000xg for 5 min), the precipitated fraction was placed on a microscope slide and sealed. The CR birefringence was detected under cross-polarized light using an optic microscope (Leica DMRB, Heidelberg, Germany).

### Thioflavin-T Assay

Th-T binding to the purified protein fractions of bacteria and plant leaves was recorded using a Varian spectrofluorometer (Cary Eclipse) (Varian Inc., Palo Alto, CA, USA) with a excitation wavelength of 445 nm and emission range from 470 nm to 570 nm and the emission at 480 nm was recorded. Final Th-T and protein concentrations were 25 µM and 0,04 mg/ml in BUFFER, respectively. Spectra were recorded after 2 min equilibration and solutions without protein were used as negative controls. Binding of Th-T to a 10 µM amylin amyloid fibril solution was used as positive control. For microscopy analysis, proteins were incubated for 1 h in presence of 125 µM of Th-T. After centrifugation (14 000xg for 5 min), the precipitated fraction was placed on a microscope slide and sealed. Th-T relative fluorescence images of purified IBs were obtained at 40-fold magnification under UV light in a Leica fluorescence DMBR microscope (Leica Microsystems, Mannheim, Germany).

### Electronic transmission microscopy

The purified protein fractions of bacteria and plant leaves where diluted ten fold in water, placed on carbon-coated copper grids and left to stand for five minutes; then, the grids were washed with distilled water and stained with 2% (w/v) uranyl acetate for another two minutes before analysis using a HitachiH-7000 transmission electron microscope operating at an accelerating voltage of 75 kV.

### Prediction of aggregation-prone regions

Two different algorithms were used to predict the potential presence of aggregation-prone regions in the sequence of transglutaminase: TANGO (http://tango.crg.es), which is based on the physicochemical principles underlying ß-sheet formation, extended by the assumption that the core regions of an aggregate are fully buried and AGGRESCAN (http://bioinf.uab.es/aggrescan/) which uses an aggregation-propensity scale for natural amino acids derived from in vivo experiments. The default prediction parameters were used for both programs.

### Preparation and aggregation of a C-terminal peptide of TGZ

The peptide QLVVLDILLGKFS corresponding to residues 465–477 of maize TGZ was obtained from EZBiolab Inc. (Carmel, IN, USA) with a purity of 92.62%. Stock solutions were prepared at 5 mM in 1,1,1,3,3,3-hexafluoro-2-propanol (HFIP), then centrifuged at 15 000 g at 4°C for 15 min and finally were filtrated through millex-GV 0.22 μm filters in order to avoid the presence of pre-aggregated species in the assays. HFIP was removed by evaporation under a gentle stream of nitrogen and the samples were stored at −80°C until analysis. The peptide was resuspended in 1 mL of 50 mM TRIS at pH 7.5, 150 mM NaCl at 4°C at 100 µM and the samples bathsonicated for 5 min previous to aggregation assays. The peptide aggregation was carried out at 25°C without and with agitation (500 rpm) for 48 h and amyloid properties evaluated as described above.

### Western blot analysis

Proteins of the soluble and insoluble fraction were quantified by the Bradford method using the Bio-Rad© reactive. Proteins were separated by SDS-PAGE in a Mini-Protean III system (Bio-Rad, Hercules, CA, USA) adding 3 mg protein per ml of buffer in the presence or absence of 8 M Urea. About 100 µg total protein was added per well. Separated proteins were further transferred to nitrocellulose membrane (GE Healthcare, Little Chalfont, UK) on wet system (Bio-Rad, Hercules, CA, USA) according to manufacturer's instructions. Membrane blocking was performed with non-fat dry milk (5%, w/v) in PBS 1× and washed in PBS with Tween 20 (0.1 to 0.3%, v/v). Immunodetection was carried out by using a polyclonal antibody raised against maize TGZ protein expressed and purified from *E. coli* [18] as primary antibody diluted 1/5000, and a peroxidise-conjugated goat anti-rabbit IgG (AO545, Sigma-Aldrich, Spain) at 1/15000 dilution as the secondary antibody. Immunodetection was obtained by chemiluminescence (ECL, Amersham Pharmacia Biotech©) following the manufacturer's instructions. Western-blot images were acquired in a LAS-3000 Fuji (Japan) Imaging System.

## Supporting Information

Figure S1 Prediction of aggregation prone regions in maize TGZ sequence. A) Amino acid sequence of TGZ. The regions with the highest predicted aggregation propensities are shown in red. B) AGGRESCAN aggregation profile of the 150 C-terminal residues of TGZ (residues 1 and 151 in the profile correspond to residues 384 and 534 in TGZ, respectively). B) TANGO aggregation profile of the 150 C-terminal residues of TGZ (residues 1 and 151 in the profile correspond to residues 384 and 534 in TGZ, respectively).
(0.20 MB PDF)
