# Supplementary material for: Amyloid-Like Protein Inclusions in Tobacco Transgenic Plants
Source: PLoS One. 2010 Oct 26;5(10):e13625. doi: 10.1371/journal.pone.0013625 (PMC2964307; doi:10.1371/journal.pone.0013625)

A

MAHRGHLDGL TGQAPALMRH GSFAAGSLSS RSPLQSSSTL EMLENKLAMQ TTEVEKLITE
NQRLASSHVV LRQDIVDTEK EMQMIRTHLG EVQTETDLQI RDLLERIRLM EVDIHSGNVV
NKELHQMHME AKRLITERQM LTLEIEDVTK ELQKLSASGD NKSLPELLSE LDRLRKEHHN
LRSQFEFEKN TNVKQVEQMR TMEMNLITMT KQAEKLRVDV ANAERRAQAA AAQAAAHAAG
AQVTASQPGQ LKLPRFQQQQ PQTHMQVHIP ATPLHISREP RLGHISRVLR LGYISREPRL
GHISREPRLG HISRGARMGH ISRGLRLGHI SREPRLGHIS REPRLGHISR VLRLGHISRE
PRLGHISRGP SLGHISRGPR LGHISREPRM GHISREPRMG HISRVLRLEH TTMLMMLARL
MHMQVTLAIQ LQATRKVQCP TIPMLHLRSQ QAAVQLRTPQ EASMGQLVVL DILLGKFSRA
VALQMQRKHL LLHHHRQHHI PPAHMTKPEE PRDKIWDVNQ MDVCHAHLLS RQIW

B

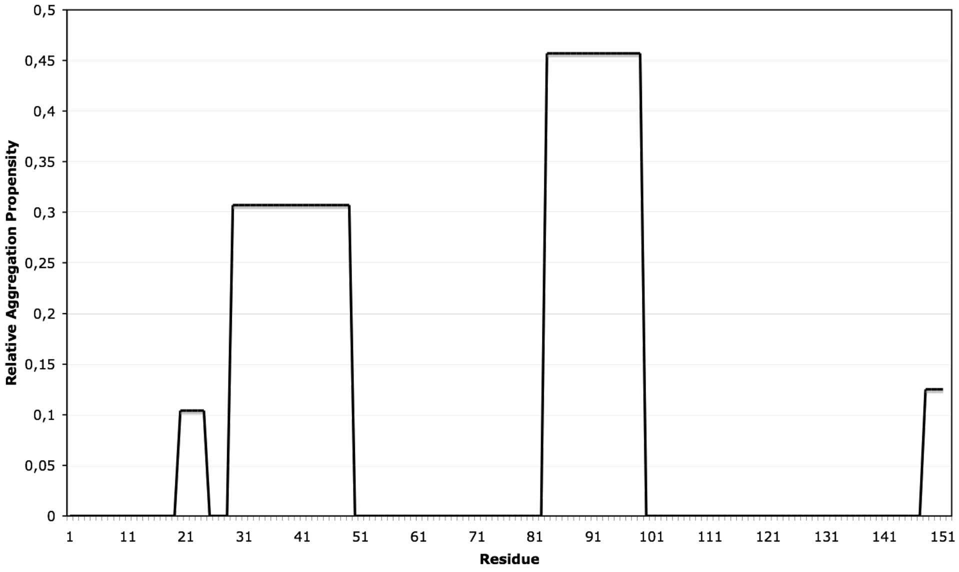

C

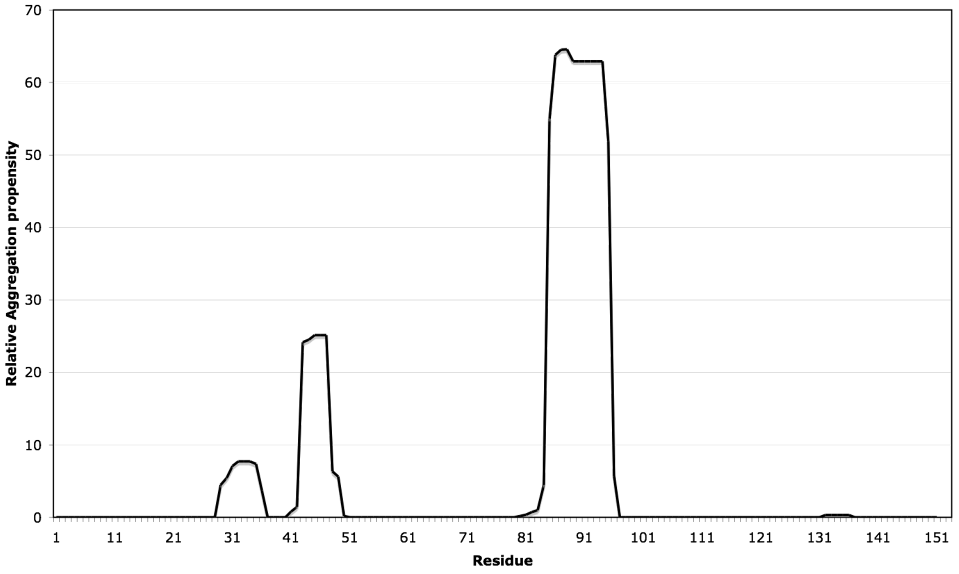

Supplement: Figure S1 — Prediction of aggregation prone regions in maize TGZ sequence. A) Amino acid sequence of TGZ. The regions with the highest predicted aggregation propensities are shown in red. B) AGGRESCAN aggregation profile of the 150 C-terminal residues of TGZ (residues 1 and 151 in the profile correspond to residues 384 and 534 in TGZ, respectively). B) TANGO aggregation profile of the 150 C-terminal residues of TGZ (residues 1 and 151 in the profile correspond to residues 384 and 534 in TGZ, respectively). (0.20 MB PDF) [file pone.0013625.s001.pdf]
